# Transcription factor-based biosensors for screening and dynamic regulation

**DOI:** 10.3389/fbioe.2023.1118702

**Published:** 2023-02-06

**Authors:** Jonathan Tellechea-Luzardo, Martin T. Stiebritz, Pablo Carbonell

**Affiliations:** ^1^ Institute of Industrial Control Systems and Computing (AI2), Universitat Politècnica de València (UPV), Valencia, Spain; ^2^ Institute for Integrative Systems Biology I2SysBio, Universitat de València-CSIC, Paterna, Spain

**Keywords:** allosteric transcription factors, biosensors, screening, dynamic regulation, metabolic engineering

## Abstract

Advances in synthetic biology and genetic engineering are bringing into the spotlight a wide range of bio-based applications that demand better sensing and control of biological behaviours. Transcription factor (TF)-based biosensors are promising tools that can be used to detect several types of chemical compounds and elicit a response according to the desired application. However, the wider use of this type of device is still hindered by several challenges, which can be addressed by increasing the current metabolite-activated transcription factor knowledge base, developing better methods to identify new transcription factors, and improving the overall workflow for the design of novel biosensor circuits. These improvements are particularly important in the bioproduction field, where researchers need better biosensor-based approaches for screening production-strains and precise dynamic regulation strategies. In this work, we summarize what is currently known about transcription factor-based biosensors, discuss recent experimental and computational approaches targeted at their modification and improvement, and suggest possible future research directions based on two applications: bioproduction screening and dynamic regulation of genetic circuits.

## 1 Introduction

Biosensors are biological devices combining two essential components: a sensing component that detects a particular input—typically the presence of a chemical—and a reporter that produces a measurable output after receiving the signal transduced by the sensing component. Whole-cell biosensors use biochemical transformations inside living cells to detect and react to different inputs (Fernandez-López et al., 2015).

One important class of whole-cell biosensors are those based on transcription factors (TF). TFs are proteins that can control the expression of genes by binding to specific DNA sequences. Some TFs are triggered after binding to a metabolite or external compound (known as allosteric transcription factors, aTFs). Once activated, a conformational change in the TF makes itself release from or attach to the DNA sequence upstream of the target gene, thereby activating or repressing its expression. TFs can be assembled together with other DNA parts commonly used in synthetic biology, such as promoters, ribosome binding sites (RBSs), terminators and reporter genes, to create TF-based biosensor circuits. These genetic devices can thus be used to sense and react to a range of intracellular or environmental ligand concentrations (De Paepe et al., 2018). Even though allosteric transcription factors are suitable building blocks for the design of biosensors, they might require prior optimization or changes to their ligand specificity. The literature contains several examples in which sophisticated directed-evolution strategies were applied to this end (Wu et al., 2017; Machado et al., 2019; Berepiki et al., 2020; Snoek et al., 2020), and we will discuss this approach in more detail in Sections 2, 3.

aTFs can present several architectures. The relationship between the effector molecule and the aTF defines their mode of action: repression of activator aTF, activation of repressor aTF, repression of repressor aTF, or activation of activator aTF (Mannan et al., 2017). A sizable number of aTFs have been found for each category, allowing one to build biological circuits with a large variety of complex functions. However, the use of TF-based biosensors in complex applications such as the industrial scale-up of bioproduction processes or intricate biocomputing circuits has been stalled. This is mainly due to the fact that the number of metabolite-activated TFs which have been described in the literature (Koch et al., 2018) is rather small compared to the large number of compounds potentially amenable to biomanufacturing. Additionally, biosensing circuits often perform poorly due, for example, to non-specific activity, cross-talk with native biochemical reactions, leaky expression and problematic or impossible heterologous expression. It is therefore becoming increasingly clear that the number of engineered TF-biosensors and the means for their optimisation need to keep up with the growing demands of the synthetic biology community.

Here, we provide a roadmap for the design of new biosensor circuits based on aTFs that leads from gathering data and theoretical prediction to experimental validation. We also provide guidelines for the rapid prototyping of biosensor circuits with improved features using computational tools and discuss experimental validation methods best suited for this task. Additionally, we focus on two crucial applications of biosensors for current synthetic biology targets, namely production screening and dynamic regulation.

## 2 Determining the design space of detectable compounds and aTFs

### 2.1 Exploring the current knowledge of the biosensor space

For years, researchers have studied the regulatory networks of different cells and organisms to understand, among other things, how they react to environmental changes by controlling essential cellular activities through the expression or repression of their genes. With the advent of genetic engineering and synthetic biology, this information can nowadays be used to re-engineer and create fine-tuned genetic circuits for various purposes, notably biosensors. However, the data is often scattered and incomplete, and gathering efforts have to be made to organize and make easily available the current knowledge on the topic. Through literature and database mining, it is possible to build a dataset of transcription factors triggered by the binding of molecules as well as other types of inputs (temperature, light, pH…). This valuable dataset can be used to determine the known detectable input space (Koch et al., 2018), i.e., the set of molecules that can be detected by TF-based biosensors. Table 1 describes the main databases that can be used to generate this space. Note that the databases might gather different types of data: TF gene sequence, binding sites, ligands, TF regulated genes and/or structure, thus restricting data integration.

**TABLE 1:** Name, description, bibliographic reference, and web link to some of the most important TF databases. The description information was directly taken from the website.

Databases	
Name and description	References
P2TF (Predicted Prokaryotic Transcription Factors): an integrated and comprehensive database of TF proteins, which contains a compilation of the TF genes within completely sequenced genomes and metagenomes	Ortet et al. (2012)
JASPAR: an open-access database of curated, non-redundant transcription factor (TF) binding profiles stored as position frequency matrices (PFMs) and TF flexible models (TFFMs) for TFs across multiple species in six taxonomic groups	Castro-Mondragon et al. (2022)
TF2DNA: database provides comprehensive information about transcription factor binding motifs and their regulated genes for five model organisms and humans	Pujato et al. (2014)
GRASSIUS: Divided in GrassTFDB which provides a comprehensive collection of transcription factors from maize, sugarcane, sorghum and rice and GrassCoRegDB which provides a collection of proteins that are transcriptional regulatory factors but do not bind DNA in a sequence specific fashion	Yilmaz et al. (2009)
RegulonDB: the primary database on transcriptional regulation in *Escherichia coli* K-12	Santos-Zavaleta et al. (2019)
SM-TF database: collects available 3D structures of small molecule-transcription factor complexes from Protein Data Bank (PDB)	Xu et al. (2016)
CollecTF: a database of transcription factor binding sites (TFBS) in the Bacteria domain	Kılıç et al. (2014)
AnimalTFDB3: a comprehensive database including classification and annotation of genome-wide transcription factors (TFs), and transcription cofactors in 97 animal genomes	Hu et al. (2019)
PlantTFDB: Plant Transcription Factor Database	Jin et al. (2017)
RegPrecise: a database for capturing, visualisation and analysis of transcription factor regulons that were reconstructed by the comparative genomic approach in a wide variety of prokaryotic genomes	Novichkov et al. (2013)
SigMol: a repertoire of Quorum Sensing Signalling Molecules in Prokaryotes	Rajput et al. (2016)
Bionemo: stores manually curated information about proteins and genes directly implicated in the Biodegradation metabolism	Carbajosa et al. (2009)
PRODORIC: a comprehensive database about gene regulation and gene expression in prokaryotes. It includes a manually curated and unique collection of transcription factor binding sites	Dudek and Jahn (2022)
Tools	
footprintDB: predicts transcription factors which bind a specific DNA site or motif and DNA motifs or sites likely to be recognized by a specific DNA-binding protein	Sebastian and Contreras-Moreira (2014)
CiiDER: a user-friendly tool for predicting and analysing transcription factor binding sites, designed with biologists in mind	Gearing et al. (2019)
BART (Binding Analysis for Regulation of Transcription): a bioinformatics tool for predicting functional transcriptional regulators (TRs)	Wang et al. (2018)
PROMO: a virtual laboratory for the identification of putative transcription factor binding sites (TFBS) in DNA sequences from a species or groups of species of interest	Messeguer et al. (2002)
DeepTFactor: a deep learning-based tool for the prediction of transcription factors	Kim et al. (2021)

The databases in Table 1 can be considered as good starting points when compiling the list of known ligand-responsive TFs. However, some may not have been recently updated, and thus some important bits of information may be missing. In order to expand the initial set, Natural Language Processing (NLP) may be used to mine additional information from the literature helping to fill in the knowledge gap of the metabolite detectable space, i.e., the set of metabolites that can be detected through TFs-based biosensors. Bibliographic databases such as NCBI are ideal sources of input data for this type of text mining algorithm. NLP has successfully been used in other biotechnology applications, as, for example, in the context of predicting protein-protein interactions and establishing gene-disease relationships (Zeng et al., 2015), and could also be used to find aTF-ligand interactions.

### 2.2 Homology-based prediction to enlarge the aTFs dataset

**FIGURE 1:**
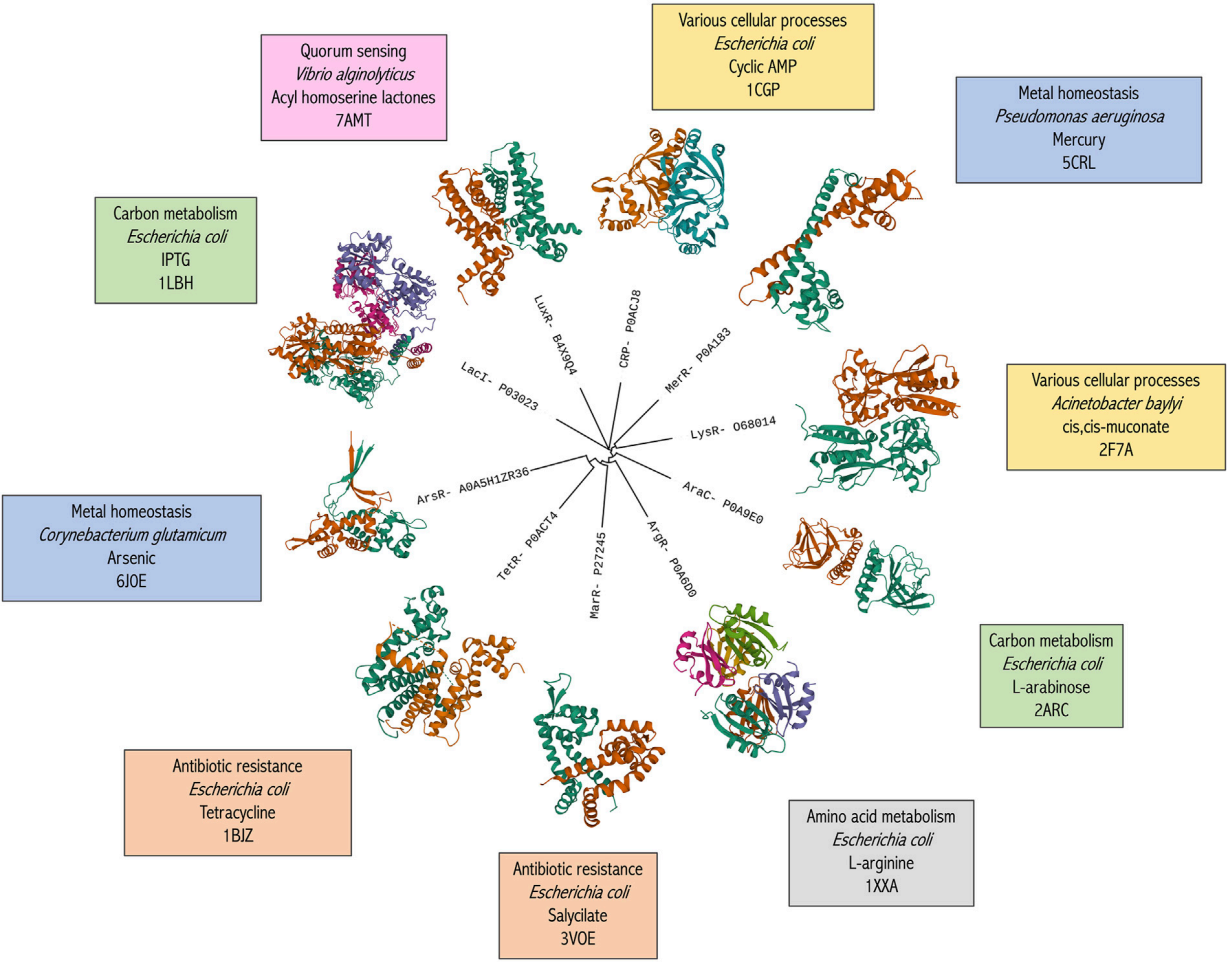
Broad phylogenetic classification of the ten most common bacterial aTF families. Family name and representative UniProt IDs are used as tree labels. Structure images were obtained using Mol* Viewer (Sehnal et al., 2021) *via* RCSB PDB. The text boxes detail, in order, the most common aTF-controlled pathway, the species name of the specific aTF, the representative effector molecule and the PDB identifier of the structure.

The body of experimental data on aTFs, which is currently available in a curated form, can also be extended through homology-based prediction of TF sequences in other species (Figure 1). Using protein sequence information of well-known transcriptional regulator families (e.g., LysR, TetR, AraC, *etc.*) (Fernandez-López et al., 2015) as a reference, annotation experiments can be made on target genomes that are publicly available (Deplancke and Gheldof, 2012). Similarly, metagenomes can be mined (Oliveira Monteiro et al., 2021) to find more information about, for example, non-culturable species. Some of the databases described in Table 1 were built, either in their entirety or in part, by using genomic or metagenomic data.

Protein structure information can also be used to discover and engineer new biosensors. However, despite the rather large number of protein structures available for certain aTFs (e.g., TetR, TrpR, AraC), many candidates still await structural characterization, a problem that negatively impacts the development of biosensors tailored to recognize arbitrary compounds of interest. Although many aTFs do show sufficient homology with existing structures and might therefore be targeted with homology modeling, the quality of these models can at times be lacking—especially in the twilight zone of sequence homology. This, in turn, limits the accuracy with which residues that are crucial for the binding of effector candidates can be predicted by docking calculations. Based on the current knowledge of aTF-ligands (Koch et al., 2018), we calculated that not more than 45% of the TFs in this dataset share more than 50% of identical residues with experimentally solved protein structures.

### 2.3 AI-based prediction of new aTFs

Traditional homology and structural-based methods are not the only approaches that can be used to predict novel TFs. In recent years, as in other biotechnology fields, AI-based applications have been widely adopted (Volk et al., 2020). The predictive power of AI can be exploited by combining the information available in the aforementioned databases with reference genomic databases such as NCBI, to train the algorithms for predicting new TFs. In 2021, Kim and others presented DeepTFactor (Kim et al., 2021), which was able to predict over 300 TFs in the well-studied *Escherichia coli* K-12 genome, including TFs not previously reported in databases, and which the authors were able to validate using TF and non-TF protein sequences as training data. This tool can be used to identify new TF sequences from known and new genomic and metagenomic information, in combination with sequence-based annotation tools. Other AI-based methods that identify DNA binding protein domains (Eichner et al., 2013; Mishra et al., 2019; Li et al., 2021) from sequences may also be considered for this purpose. The recent progress in the application of deep learning to the *de-novo* prediction of nucleic acid/protein complex structures, such as RoseTTAFold2NA (Baek et al., 2022) and DeepFoldRNA (Pearce et al., 2022), might make it possible to structurally validate TF/DNA complex formation and binding for those TF candidates that show no or only poor homology with known protein structures.

Protein structures open an extra layer of useful data for researchers trying to complete the atlas of aTFs. In a milestone research article, AlphaFold was published in 2021 as a Machine Learning prediction tool, able to predict protein structure using only the protein amino acid sequence as input (Jumper et al., 2021). This tool is expected to provide reliable and fast structure information that would otherwise take years to be resolved through experimental methods.

**FIGURE 2:**
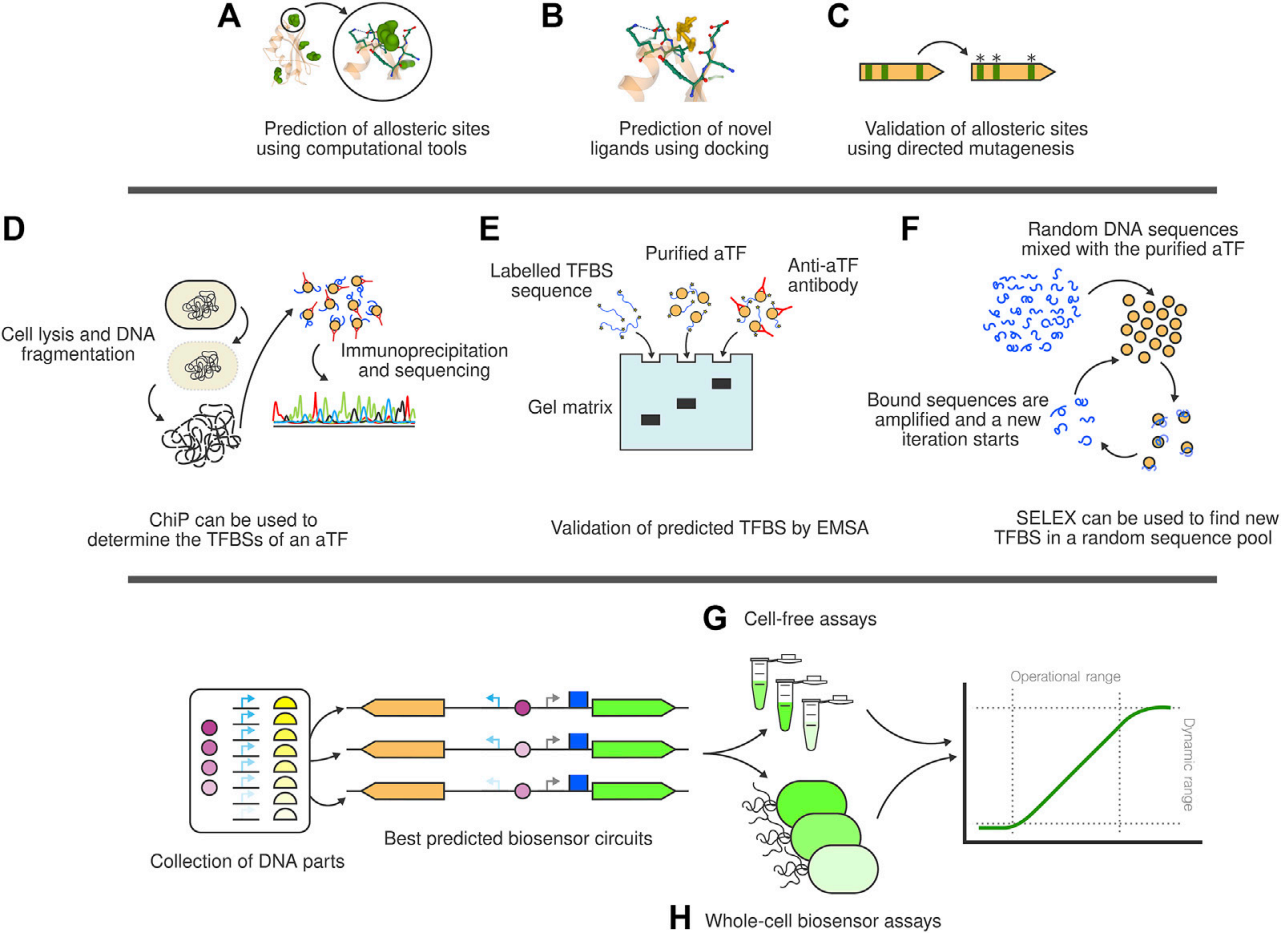
The full stack biosensor development toolbox. **(A)** Computational tools can be used to determine the allosteric pockets of interaction between ligands and aTF. **(B)** Using these pockets as reference, docking computations can be carried out to assess the affinity of the aTF towards a library of putative ligand compounds. **(C)** The allosteric site computation can be validated using directed mutagenesis to evaluate changes in affinity or specificity. **(D)** ChIP technology allows researchers to determine the TFBSs of a newly discovered aTF. **(E)** Similarly, EMSA can be used to individually validate single TFBS. **(F)** SELEX can be used to artificially obtain new TFBS to the aTF. **(G)** Cell-free assays are a quick prototyping technology to test biosensor circuits once assembled. **(H)** Whole-cell biosensor experiments allow the characterisation of the biosensor circuit in conditions closer to *in vivo* applications.

Once a putative TF has been identified, determining the TF binding site (TFBS) (i.e., the DNA region where the TF attaches to/detaches from) is the next step. Sequence homology among TFBSs of the same aTF can be used together with AI (Koo and Ploenzke, 2020; Chen et al., 2021) to predict new TFBSs. Some of the databases in Table 1 (Santos-Zavaleta et al., 2019; Castro-Mondragon et al., 2022) provide this information for each of their entries. To that end, a structural component that determines the activity of an aTF is its ligand binding domain (LBD). The practical applicability of TF-based biosensors is limited by the relatively small pool of known ligand binding domains. Designing and experimentally validating new binding domains has been a long-standing challenge. Thanks to continued work and recently introduced algorithms (Lucas and Kortemme, 2020; Polizzi and DeGrado, 2020), a viable solution seems now within reach but not yet at hand. In addition to high ligand affinity and specificity, the receptor system must be activated once bound to the effector molecule, going through some form of conformational change in order to trigger the biosensor response (Su and Hammond, 2020). This activation can be hard to engineer for new molecules and protein domains. AI might also be able to address this challenge, as it can be used not only to identify new putative TFs, but also to predict the pockets (i.e., sites) of allosteric interaction (Figure 2A) with the ligand in the LBD (Panjkovich and Daura, 2014; Greener and Sternberg, 2015; Xiao et al., 2021).

Once novel TFs and their TFBSs have been found, it is necessary to determine their most likely effector molecules. To do this, Hanko and others (Hanko et al., 2020) described an approach to identify whole metabolite-inducible systems (i.e., TF, inducible promoter and its corresponding effector). The method looks at the operon next to the predicted TF and assigns the effector molecule as the primary substrate metabolized by the operon-encoded enzymes. A structure-based solution was also made available in (Huang et al., 2018) where Huang et al. presented a computer tool that finds allosteric sites inside a structure and calculates a docking score for each molecule in a database (Figure 2B).

Allosteric sites can be good candidates for AI-mediated site-directed mutagenesis experiments (Cadet et al., 2018; Saito et al., 2018; Wu et al., 2019) (Figure 2C) trying to improve the specificity of the TF towards the ligand or adapting its affinity towards novel molecules. Machine Learning-centered structural approaches such as AlphaFold might soon lead to a breakthrough in the field of transcription factor design, in particular because the most recent iteration, AlphaFold-Multimer (Evans et al., 2021), is able to produce multimeric solutions. These can be especially relevant for the effector binding domains (EBDs) of homodimeric aTFs and are a prerequisite for predicting binding modes of effector molecules accurately. Of note are also very recent language-model-based approaches that appear to be computationally more efficient and are able to predict multimeric states even though they have not explicitly been trained on protein complexes (Lin et al., 2022).

The design process of bespoke binding domains for non-cognate ligands would also benefit from the development of faster and more reliable *in silico* docking algorithms. Recent ML-based advances, such as DiffDock (Corso et al., 2022), might provide a crucial advantage over classic methods in this context. The computational speed-up promised by these approaches might also make it possible to explicitly model water molecules and thereby further increase the predictive quality of the docking process, because water-mediated interactions often play a crucial role in ligand binding sites. The prediction of binding modes for possible effector molecules is necessary but not sufficient for the reliable computational design of biosensors due to the delicately tuned allosteric properties of aTFs. These can make protein engineering attempts to alter ligand-binding specificity quite challenging, given the possibility of negatively impacting signal transduction.

Unfortunately, predicting the dynamic properties of an aTF upon ligand binding and/or mutation is significantly more challenging than calculating binding modes and usually involves long time-scale molecular dynamics simulations. This problem could, in principle, be addressed with coarse-grained molecular dynamics methods that approximate fully atomistic simulations, such as the latest iteration of the Martini model, which has successfully been applied to ligand binding (Souza et al., 2020). Crucial for its applicability, however, is the availability of parameters for the ligands to be studied. For the Martini model, this issue is being addressed by the creation of a curated ligand database and the development of automated tools for the generation of coarse-grained models (Souza et al., 2020; Hilpert et al., 2022). It is, however, conceivable that the problem of predicting ligand-induced conformational changes in aTFs can be addressed with deep-learning approaches as well, which could constitute a breakthrough for the *in silico* component of biosensor-engineering.

### 2.4 Experimental validation of predicted biosensors

Once a putative new aTF has been identified, experimental validation is necessary to check its DNA binding site, affinity towards different ligands, and performance. For each of these tasks, some validation procedures have been defined. Some of the tools described in previous sections and in Table 1 can be used to predict TF DNA binding sites. However, for the subsequent development of a functioning biosensor circuit, it is crucial to experimentally confirm that the TF actually binds to the effector molecule and to its target DNA sequence.

For instance, chromatin immunoprecipitation (ChIP) is commonly used to assess the binding sites of the aTF anywhere in the genome, effectively assessing the regulated genes (Figure 2D). To this end, the cells are lysed, the genome is fragmented and TF-DNA complexes are isolated thanks to TF-specific antibodies (van Werven, 2006; Grainger et al., 2007). In a further step, the DNA in the complex can be sequenced to determine the TFBS.

The Electrophoretic mobility shift assay (EMSA) is a standard procedure that can be used to confirm the DNA binding site of newly discovered TFs (Alves et al., 2021; Modrzejewska et al., 2021) (Figure 2E). Basically, the purified TF is mixed with a labelled DNA probe of the putative binding sequence and ran in an agarose gel. The TF-DNA complex runs slower compared to the free DNA sequence (Gurevich et al., 2010). EMSA, however, is not scalable. Other procedures have been used to determine, using high-throughput technologies, the DNA binding sequences of hundreds of TFs annotated in a species. Wang and others (Wang et al., 2021), for example, determined the binding specificities of 182 TFs of *Pseudomonas aeruginosa*. To do this, they purified 371 putative TFs, mixed them with randomised DNA sequences and ran high-throughput systematic evolution of ligands by exponential enrichment (HT-SELEX) (Figure 2F) for four cycles. Both EMSA and the HT-SELEX require the TF to be previously purified. This can quickly become a bottleneck. For this reason, a bacterial one-hybrid system was described in 2005 (Meng et al., 2005). In a nutshell, this procedure allows researchers to assess the binding specificities of TFs against random DNA sequences *in vivo* by linking the TF-DNA binding with the expression of positive and negative selection markers. This method does not require proteins to be purified or the availability of antibodies and provides a low-tech scalable method of finding DNA binding sequences.

### 2.5 Extending the biosensor space through bioretrosynthesis

In the cases where no aTFs are known to be triggered by the target compound, alternative approaches may be considered. One possibility is to enlarge the biosensor space is through retrosynthesis approaches (Delépine et al., 2016). The approach enables modulation of the specificity and dynamic range of the biosensor by introducing metabolic conversions as part of the sensing process. In this way, the number of targets that can be detected can be substantially increased since any chemical target that can be converted into a molecule for which an aTF exists becomes potentially detectable. In order to compute the metabolic pathways that can connect the target to existing aTFs, bioretrosynthesis-based approaches are used (Lin et al., 2019). Such algorithms generate a tree-like graph of biochemical conversions connecting the target to those molecules that can be detected. SensiPath (Delépine et al., 2016) is an online server that can compute the alternative extended aTF-based biosensors for any given target.

This approach has been used for instance to analyse the biosensing of the production of naringenin that is used for dynamic regulation through its conversion into kaempferol (Boada et al., 2020). The study showed how the dynamic range of the resulting biosensor could be adjusted up to industrial levels of 1 g/L in a bioreactor, in a way that would have been more challenging by using direct biosensing of naringenin.

The approach has also been used systematically in order to develop a protocol for the development of cell-free biosensors through metabolic and genetic layers (Soudier et al., 2022). The authors proposed a standard methodology based on computer-aided design (CAD) that combined the design of a perceptron-like genetic device (Pandi et al., 2019a) with the automated selection of enzymes for the metabolic pathway through the Selenzyme algorithm, an online tool that suggests best candidate enzyme sequences based on the biochemical conversions in the pathway (Carbonell et al., 2018a).

Other engineering efforts have been showcased which focus on different areas. Biocomputing, for instance, would also benefit from a wider range of TF-based biosensors. To that end, Rondon and others (Rondon et al., 2019) created 27 new synthetic TFs starting from 6 core TF domains, 7 DNA recognition domains and 7 operator regions that are able to detect 5 different ligands.

## 3 Biosensor design

### 3.1 Biosensor library characterization and fine-tuning

The predictions and the *in vitro* and *in vivo* validation experiments provide essential information to biosensor designers. An intermediate step between prediction and experimental validation and actual whole-cell biosensor construction can be achieved by cell-free assays. The technique relies on cell extracts containing all the necessary cellular components for protein expression. It removes cellular maintenance, growth, other native processes and all cellular unknowns from the equation, allowing the experiment to be essentially focused on the synthetic circuit and behavior designed by the researchers (Hodgman and Jewett, 2012). Cell-free assays (Figure 2G) provide a simple and standardizable approach to quickly test biosensor genetic circuits (aTF, aTF promoter, reporter, operator…) *in vitro*. Examples of successful cell-free biosensors have been described, that detect chemicals such as quorum sensing molecules (Wen et al., 2017), rare sugars such as D-psicose (Pandi et al., 2019b) and water contaminants (Jung et al., 2020), among others.

However, building a biosensor circuit that works inside the cell (Figure 2H) and that provides a measurable output is the litmus test of the successful functionality of the new aTF. In its most basic form, a biosensor circuit will express the aTF and a reporter (e.g., a fluorescent protein). The latter is under the control of a promoter containing the DNA-binding sequence recognized by the TF. The binding of the effector molecule with the aTF regulates gene expression of the reporter gene. Some considerations to bear in mind during the design of such metabolite-responsive biosensors, specifically for the aTFs, can be found in (Liu et al., 2017).

Once a circuit has been designed and built, the next step is to determine how well it works under a range of conditions; that is, to characterise the biosensor. Characterisation experiments determine, among other parameters, the dynamic range, the operational range and the dynamic response of the biosensor (Mannan et al., 2017). It is likely, however, that the initial biosensor design is not fit for its intended purpose and tweaks need to be done in the circuit for the biosensor’s performance to meet the designer’s criteria (e.g., dynamic range, operational range, specificity, speed of response…). The dynamic and operational range of the biosensor can be engineered by changing the expression levels of both the TF and the reporter gene through RBS (De Paepe et al., 2018) or promoter (Sonntag et al., 2020) engineering. The number and position of the aTF operator region(s) can also be used to modulate the dynamic range of the biosensor (Xu et al., 2020). On the other hand, the generation of chimeric aTFs, obtained by merging the DNA binding domains and ligand binding domains from different genetic sources has also been successfully tested to modulate the specificity towards other ligands (De Paepe et al., 2019). A similar approach based on the high-throughput fusion of periplasmic binding proteins and DNA binding domains was presented in Juárez et al. (2018). A complete overview of the different approaches that can be used to fine-tune the initial biosensor was provided by De Paepe et al. (2017).

Apart from poor inherent behaviour of the aTF, the compatibility of the heterologous host with the transcription factor is often the main culprit when the performance of the newly generated biosensor is inadequate. The portability of genetic circuits between species has been challenging synthetic biology since its early stages. In bioproduction projects, it may be possible that the best host for the production of the target compound is not suitable for the expression of the biosensor circuit. In the native species, transcriptional regulation often implies a complex regulatory system composed of several membrane transporters, inhibitors, activators and cofactors (Carpenter et al., 2018). For well characterised regulators, these may be known but newly found aTFs may have unknown necessary components. The challenge is bigger when the aTF system is transferred from phylogenetically distant species (e.g., from plants to bacteria). Steps that can be taken to improve the performance of biosensor circuits include: optimization of gene expression, selection of an adequate reporter system (see next Section) and the incorporation of additional genetic modules which may entail the addition of compound importers, exporters, leak dampeners and other types of signal modulation devices (e.g. amplifiers, inverters…) (Miller et al., 2022).

### 3.2 Biosensor reporter selection

An important and often overlooked factor in designing and building new biosensor circuits is the selection of the reporter gene. Fluorescent reporters are the most common choice. Classic fluorescent proteins (FPs) such as GFP and RFP are simple to assemble in genetic circuits, easy to measure and do not rely on any metabolic substrate (other than oxygen) to work. Most of the experimental references detailed in this work use FPs as reporter systems and this type of biosensor is predominantly used in the field. Different FPs offer different characteristics to the biosensor designer. One should consider, among other factors, the excitation and emission wavelength, the maturation time (Shaner et al., 2005) and the half-life of the matured protein [as the FP can sometimes be too stable and rendered useless in real-time applications such as biosensing (Andersen et al., 1998)]. Bioluminescent proteins are other alternative reporters (Nourmohammadi et al., 2020; Hansen et al., 2021) that rely on biochemical reactions emitting photons as products. Compared to fluorescent proteins, bioluminescent reporters do not rely on the measuring equipment to excite a fluorophore, which leads to less background emission and higher sensitivity.

Nevertheless, there are other alternative reporters that offer different features that may be more appropriate for specific purposes. Before fluorescent and luminescent signal detection equipment became ubiquitous in molecular biology laboratories, colorimetry was often the most efficient way to detect biological processes using the naked eye or simple absorbance measurements. Biosensors have been developed using the colorimetric reporters lacZ (Choi et al., 2013; Li et al., 2017; Hansen et al., 2021) and the carotenoid pathway (Yoshida et al., 2008; Watstein et al., 2015). In recent years, the violacein pathway has gained popularity as a tunable route where each intermediate compound can act as a measurable reporter (Watstein et al., 2015; Hui et al., 2020; Guo et al., 2021). Reporters that utilize electrical signals have also been proposed and are derived from electrogenic bacteria (Golitsch et al., 2013; Webster et al., 2014; Zhou et al., 2017) or are obtained *via* a synthetic electron transport chain (Atkinson et al., 2022), where the generation of an electric current allows for faster responses and actuation than can be achieved with protein expression-based systems.

A thorough consideration of the features and issues of each reporter category should be taken into account before committing to a reporter. A comparison of 8 different reporters of three categories can be found in (Lopreside et al., 2019). In short, enzymatic reporters (LacZ and bioluminescent reporters) can have the fastest response and the lowest detection limit for the target metabolite which is perfect for biosensors requiring precise and quick measurements. However, these advantages require the cells to be lysed and the enzymatic substrate to be added to trigger the reporter reaction which does not allow the users to perform continuous quantification experiments. On the other hand, fluorescent proteins can present high media- and cellular autofluorescence and a slower response. Nevertheless, FPs have managed to become the first choice of many researchers thanks to, among other things, the possibility to simultaneously use multiple reporters with different emission patterns (green, red, blue…), the ease of use and the stability of the proteins.

### 3.3 Directed evolution of aTFs

Using smart design and predictions can be a good way to consolidate the design of the biosensor. However, when trying to, for instance, increase the sensitivity of the aTF towards new ligands, it may be necessary to apply directed evolution techniques on the gene sequence. An example can be found in the work of Rottinghaus and others, where new variants were engineered from promiscuous amino acid-specific TFs to specifically detect similar amino acids and neurotransmitters (Rottinghaus et al., 2022). A review on the evolvability of TF-based biosensors can be found in (Umeno et al., 2021). This may produce several hundreds of variants, and most of them may show equal or poorer performance, which requires a faster and simpler approach that quickly identifies and isolates good performers and discards variants that do not meet the designer’s criteria. Several methods have been described for this task.

Fluorescence-activated cell sorting (FACS) isolates cells based on fluorescence emission detected by a flow cytometer. This technique allows researchers to select, from a cell population, the individuals that perform best. FACS has previously been used to reduce the affinity space of an aTF library, thereby creating a sensor for L-histidine and L-arginine which is unable to detect L-lysine (Della Corte et al., 2020). Machado and others changed the specificity of a protocatechuic acid (PCA) biosensor to instead detect vanillin and 3,4-dihydroxybenzaldehyde using this technique (Machado et al., 2019).

The promoter under control of the effector molecule can be put in front of other types of markers. In Collins et al. (2006), the researchers managed after several rounds of directed evolution with error-prone PCR, to select variants of the aTF LuxR by isolating cells that survive on chloramphenicol and discarding variants that survive on carbenicillin by controlling the expression of cat gene and the bli gene (β-lactamase inhibitory protein, inhibits the *bla* gene) using the aTF binding site. The new variant managed to respond to new ligands, but no longer responded to the original effector.

Less common techniques have also been used to evolve aTFs to recognise new ligands. One example is a method called compartmentalised partnered replication (CPR) (Ellefson et al., 2014). In 2018, researchers generated a synthetic phylogeny from the aTF TrpR using this approach (Ellefson et al., 2018). Very briefly, CPR can be used to evolve DNA parts *via* coupling the allosteric effector activity to the expression of Taq polymerase *in vivo*. The variants that manage to produce larger concentrations of Taq will then be amplified using emulsion PCR.

Apart from the methods described so far, also phages have successfully been applied to evolve TFs. Phage-assisted continuous evolution (PACE), described in 2011 (Esvelt et al., 2011), can be used to continuously evolve any gene that can be coupled to pIII production in *E. coli*. pIII protein is required for phage infection. After mutagenesis, only the gene variants able to induce enough pIII production will propagate and enter the next cycle. A modification of this system was applied to the evolution of a TF (Brödel et al., 2020) that regulated the expression of another gene essential to phage propagation.

### 3.4 Automating the design of new biosensors

As we have described, building a new biosensor circuit using a new aTF requires many experimental steps from ligand and DNA affinity validation, to parametric characterisation, to fine tuning inside the heterologous host. All these steps can quickly become a bottleneck, especially if several ligands and aTFs are tested at the same time for different purposes. For this reason, high-throughput automated construction must be considered by the biosensor circuit designer. An application is showcased in (Tenhaef et al., 2021) where researchers rationally designed and characterised a library of aTF biosensors based on the Lrp regulator. The parts (promoters, RBS…) can also be engineered using automation, generating thousands of variants and combinations (Hossain et al., 2020). To that end, rapid prototyping strategies through automated Design-Buid-Test-Learn pipelines (Carbonell et al., 2018b) such as those implemented in biofoundry facilities (Tellechea-Luzardo et al., 2022) should alleviate current bottlenecks in the design of new biosensor circuits.

The discussion above focuses on general principles related to biosensor design. In the following section we will explore instructive examples from the literature that highlight practical aspects and possible limitations in current biosensor projects.

### 3.5 Three examples of biosensor design and optimization

Exemplarily, we will discuss here three biosensors that were successfully developed by employing current experimental and computational approaches and which also underline the relevance of structural insights into the aTF.

#### 3.5.1 Directed evolution strategy for biosensor engineering

**FIGURE 3:**
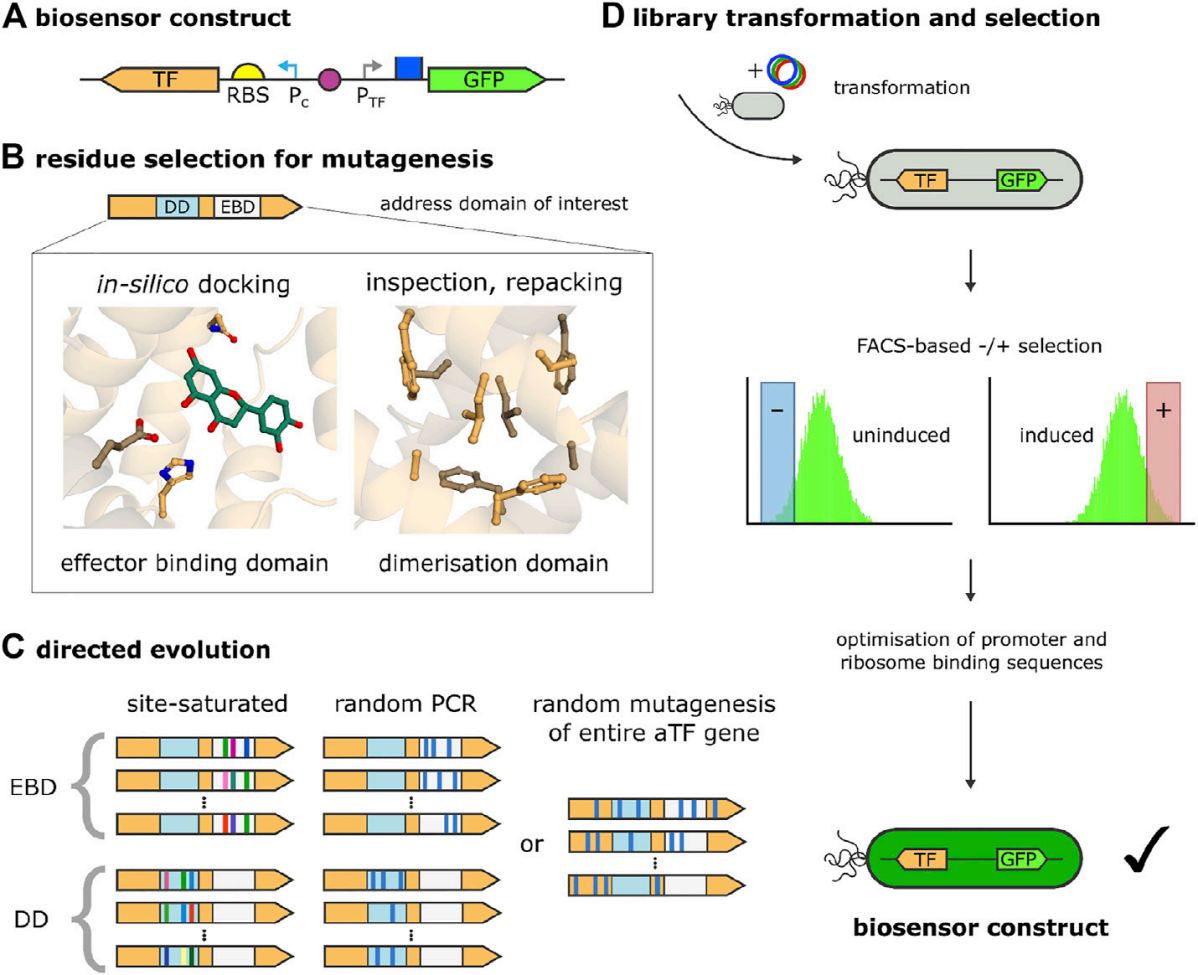
Biosensor development by directed evolution of the aTF. **(A)** Biosensor construct on one plasmid, composed of the sensor component, which, in turn, contains the transcription factor (TF) gene with a ribosome binding site (RBS) and a consecutively active promoter (P_c_), and the reporter component, comprised of a TF-inducible promoter (P_TF_) and a reporter gene (green fluorescent protein, GFP). **(B)** Biosensor mutagenesis strategies. Depending on the availability of structural information, a domain of interest of the TF (e.g., the effector binding domain, EBD, or the dimerisation domain, DD) are inspected or modeled and residues or sequence stretches for mutagenesis are selected. To guide mutagenesis, ligands can be placed into the EBD with *in silico*-docking and side chains can be designed and repacked (e.g., in the DD). **(C)** Directed evolution strategies. Based on the residues or sequence stretches selected, the domain of interest is addressed (EBD or DD in the current examples) by rational approaches, i.e., site-directed mutagenesis or random/error-prone PCR. Alternatively, the complete aTF gene is targeted with random mutagenesis in the absence of structural information, or if non-intuitive effects should be probed (e.g., those affecting allostery or DNA binding). During site-directed mutagenesis, combinations of amino acids are generated for selected, fixed sequence positions (this is represented in the picture by different colors at identical sequence positions). **(D)** The library of sensor constructs obtained by directed evolution is transformed into cells, which are then subjected to (usually) multiple rounds of negative selection (non-induced sensor in the absence of effector) and positive selection (induced sensor in the presence of effector), facilitated by fluorescence-activated cell sorting (FACS) [after (Machado and Dixon, 2022)]. The best candidates are further optimised with respect to other genetic elements determining the fidelity of the bio-sensor, such as RBS and promoter sequences.

As alluded to above, in order to alter the properties of an aTF (e.g., its ligand specificity, dynamic range, *etc.*) directed-evolution approaches have proved to be indispensable (see Figure 3 for a general overview). Directed evolution can include rational approaches which, informed by structural insight into the effector binding domain (or any other domain of interest), apply error-prone PCR within the whole region, or saturation mutagenesis to residues identified as crucial for ligand binding or other properties (Figures 3A–C). In the absence of structural or homology information, the entire protein can be subjected to random mutagenesis as well. This, however, leads to much larger strain libraries that are still unable to sample efficiently the larger sequence space, despite the higher experimental burden. An advantage of this approach is that it can reveal beneficial mutations that are less obvious than those directly affecting ligand binding, and might, among other effects, induce subtle changes in the allostery of the TF. In general, however, it is prudent to limit the size of the search space with the help of structural information, not least of all because the development of a complete bio-circuit requires the optimization of additional genetic elements, which further increases the combinatorial complexity of the design problem. This, for example, includes selecting RBS and operator sequences that perfectly harmonize with the engineered aTF.

#### 3.5.2 Development of biosensors for polyphenols

Machado et al. (2019) describe the development of a biosensor for detecting protocatechuic acid (3,4-Dihydroxybenzoic acid, PCA) by using the aTF PcaV from *Streptomyces coelicolor*. Their setup uses a two-plasmid approach, with which the expression of a GFP reporter gene is under the control of the (constitutively) expressed PcaV repressor protein. In its initial form, the biosensor was responsive to a narrow range of hydroxyl-substituted benzoate derivatives and displayed only a modest dynamic range for these effector molecules, which is commonly observed with aTFs. By applying directed evolution, Machado et al. (2019) were able to change the selectivity of PcaV so that it was able to recognize the phenolic aldehyde vanillin (4-Hydroxy-3-methoxybenzaldehyde) instead of its cognate effectors, which is quite remarkable, given the high chemical similarity between this compound and the original group of ligands. Crucial to this approach was the availability of crystal structures for PcaV, which permitted the authors to restrict the directed-evolution procedure to seven side chains that, in the PcaV protein, are in close contact with the effector PCA. To screen the strain libraries for functional mutants, the researchers developed a fluorescence-activated-cell-sorting (FACS) counter-selection protocol. This applied several rounds of negative selection (for variants that remain uninduced in the absence of the effector) and positive selection (for candidates that are also strongly inducible by the proper ligand) (see Figure 3D). Further analysis of the most promising variants revealed that a total of only three mutations sufficed to change effector recognition from PCA to vanillin and related aromatic aldehydes.

This example, in which the specificity of an aTF was successfully engineered, demonstrates the effectiveness of structure-based, directed-evolution approaches. But, as noted above, the properties of a complete aTF-based biosensor are affected by multiple variables, such as the sequences of operator and ribosome binding sites, which can turn the optimization of the complete system into a daunting task. This particular problem was addressed by Berepiki et al.( 2020) while revisiting the PcaV system just described. To this end, they merged the constitutively active pcaV gene with a GFP-reporter-gene construct on a single plasmid in order to facilitate the optimization process, which involved the systematic variation of the genetic components of the sensor, i.e., the constitutively active promoter for the expression of PcaV, the repressible PcaV-regulated promoter of the reporter gene (GFP), and the ribosome binding site for the translation of the GFP transcript. By randomizing selected positions in these genetic elements, the authors obtained three distinct sequence libraries, whose simultaneous exploration and optimization would have resulted in more than ten thousand combinations to test. In order to tame this combinatorial complexity, the researchers applied a design-of-experiments (DoE) approach which determines the ideal combination of experiments that most efficiently probe the possibility space while reducing the experimental effort. With this strategy, the authors were able to reduce the experimental setup to just thirteen distinct combinations of the genetic elements.

With the DoE approach employed, the authors assessed the impact of each variable (constitutive promoter strength of the aTF; ribosome binding site of the reporter gene; and promoter strength of the reporter gene) in a semi-quantitative way *via* statistical modeling, which even revealed non-linear effects among the parameters, and derived general design rules for repression-based aTF biosensor systems. According to the authors, the first step should be to identify the strongest combination of promoter-operator and RBS; Then, the regulator (aTF) expression should be fine-tuned by testing a wide range of expression levels. As a last step, if the dynamic range of the sensor is still not satisfactory, the RBS, which drives signal output, should be weakened.

The authors further demonstrated the generalizability of the DoE approach by optimizing a biosensor for ferulic acid that they had previously developed. With just twelve experiments, they were able to improve the maximal output signal of this biosensor by a factor of 32 and the dynamic range by a factor of five.

#### 3.5.3 Biosensor development for monitoring L-cysteine levels in cells

As discussed, structural or homology information about the aTF can be crucial for limiting the experimental effort in biosensor-design, which is also highlighted by the recent work of Gao et al.( 2022). They present the development of a novel biosensor for L-cysteine, starting from the L-cysteine-responsive transcriptional regulator CcdR. With this sensor, the authors wanted to efficiently detect and analyze cysteine-overproducing strains obtained from rational or random mutagenesis libraries. One bottleneck in directed-evolution strategies is the screening stage, for which the authors devised a high-throughput screening (HTS) method. Like the PCA biosensor discussed above, Gao et al.( 2022) developed a construct that constitutively expresses the aTF (CcdR), which, in turn, acts upon a GFP reporter gene under the control of the CcdR-specific operator ccdA. The initial construct showed proper dose-response behavior and specificity with respect to the effector L-cysteine, but low sensitivity and a narrow dynamic range, which rendered it unsuitable for the screening of large microbial variant libraries. In order to improve the initial design, the authors optimized the genetic components of the sensor system by directed evolution of CcdR, and by combinatorially optimizing promoter and RBS sequences for its expression.

For improving the properties of CcdR, the authors applied a semi-rational design strategy, made possible by the high homology of CcdR with other, structurally resolved, members of the FFRP-like (feast/famine regulatory protein) aTF family.

Because cysteine is already the cognate ligand for CcdR, the authors focused not on the effector binding but rather on the dimerization domain of the aTF, arguing that improving the dimerization properties of the repressor would increase its biological activity. Based on homology, the researchers selected eight sites in the putative dimerization interface of CcdR for saturation mutagenesis, followed by FACS-based selection, similar to the strategy described above for the PcaV-based sensor. This approach eventually led to the identification of a single-point mutant with significantly increased responsiveness to L-cysteine and a higher signal-to-noise ratio than the wild type protein. With the help of an AlphaFold-generated model (Evans et al., 2021), the authors suggest that these findings are due to improved hydrophobic interactions in the dimerization interface of the mutant.

Next, the authors tested the ideal combination from two promoter and two RBS sequence variants in order to further improve the switching dynamics and the sensitivity of the biosensor, and thus faced much less combinatorial complexity than was encountered with the approach discussed above for the PCA biosensor.

In order to facilitate the screening for L-cysteine-overproducing strains in directed evolution experiments, Gao et al.( 2022) incorporated their novel L-cysteine biosensor in an HTS platform that includes the transformation of mutagenesis libraries in cells containing the biosensor, followed by FACS-screening, colony plate screening, microplate reader analysis and finally fermentation of promising candidate clones. The effectiveness of the biosensor was demonstrated by utilizing this HTS platform in the directed evolution of L-serine-acetyltransferase, the key enzyme in the biosynthesis of L-cysteine which catalyzes its rate-determining step. By using this approach, the authors were able to directly correlate enzymatic activity with the L-cysteine levels in the cells, thereby circumventing the normal time-consuming, low-throughput sorting process. Starting from a mutant CysE library that they produced *via* error-prone PCR, they succeeded in identifying a CysE double mutant with a 7-fold increased activity, and a single mutant, whose L-cysteine producing capability was 2.7-fold higher than wild-type levels.

After demonstrating that the sensor system can in principle be applied for the screening of strains, the authors assessed its ability to handle large mutant libraries. To this end, they subjected the biosensor-containing strain to ARTP (atmospheric and room temperature plasma) mutagenesis and applied the HTS protocol, which reduced an initial amount of ten million cells to ten strains that all featured higher production levels for L-cysteine than the control.

#### 3.5.4 Development of biosensors for benzylisoquinoline alkaloids: Improving negative selection with the SELIS procedure

The previous examples relied on FACS for the selection of biosensor candidates. However, screening for strongly repressing variants by negative selection can be challenging. d’Oelsnitz et al. (2022) describe how this step can be significantly improved by suppressing the presence of dead or inactive cells, which can corrupt the cell sorting procedure. This is accomplished by making cell survival directly dependent on the repression activity of the biosensor with a method they call SELIS (seamless enrichment of ligand-inducible sensors). The approach also enables counter-selection against variants that are activated by non-target ligands, i.e., screening for repressor specificity and selectivity.

The idea behind SELIS is to add to the regular construct—consisting of the consecutive expression system for the repressor and the repressor-regulated reporter gene (see Figure 3)—One additional regulatory circuit that prevents cellular growth if the repressor only incompletely prevents gene expression in the absence of the effector. This is accomplished by growing cells in the presence of zeocin during the negative selection step, while resistance against zeocin is provided by *Sh Ble* in a way that depends on the full repression capabilities of the biosensor-candidate. To this end, the expression of *Sh Ble* is repressed by *λ* cI, which, in turn, is regulated by the same repressor-sensitive operator sequence as the reporter gene. If the repressor is fully active in the absence of an effector (or in the presence of a non-target ligand), *λ* cI cannot be formed and *Sh Ble* will be expressed, which ensures zeocin resistance and cell survival. Surviving cells can then be positively selected by plating them on zeocin-free agar plates in the presence of the effector and screening for strongly fluorescent colonies.

The authors demonstrated the effectiveness of their SELIS approach by designing sensitive and selective biosensors for a group of pharmacologically relevant benzylisoquinoline alkaloids (BIAs), namely tetrahydropapaverine, papaverine, glaucine, rotundine, and noscapine. As a starting point for biosensor development, the approach focussed on multidrug-resistance regulators that control the expression of multidrug-efflux pumps, specifically RamR from *S. typhimurium*.

The authors were able to devise an efficient mutagenesis strategy due to the availability of a crystal structure of RamR bound to the alkaloid berberine, which is structurally similar to the BIAs selected for the study. Based on this structural information, they targeted five helices surrounding the effector-binding region by creating five distinct libraries. In each library, three residues were chosen for site-saturated mutagenesis. In independent experiments, the authors also applied error-prone mutagenesis to the entire RamR gene, resulting in libraries with two mutations per gene, on average.

Starting with wild-type RamR, which displays an inherently high promiscuity for structurally diverse compounds, the authors were able with just four rounds of directed evolution, to produce highly specific biosensors that each showed >100-fold preference in binding for their cognate ligands. At the same time, each biosensor also displayed high sensitivity (<30 mM) for its target compound.

Having demonstrated the advantages of the SELIS procedure over the more traditional and purely fluorescence-based selection approach to biosensor engineering, the researchers showed how their newly evolved biosensors can be applied to the engineering of metabolic pathways. They chose the biosynthesis of tetrahydropapaverine (THP) as an example, which, in plants, involves a complicated multistep process catalysed by an oxidase and four O-methyltransferases.

Using the previously evolved THP-specific biosensor to screen for THP-producing strain variants, the authors aimed to evolve a methyltransferase from *Glaucium flavum* (GfOMT1) into an enzyme that is capable of methylating all four phenolic hydroxy groups of the substrate norlaudanosoline (NOR) in a single step to directly yield THP, thereby circumventing its complex biosynthesis and allowing for the efficient production of this pharmacologically relevant compound. To this end, they used error-prone PCR to generate mutagenesis libraries of the GfOMT1 gene, which resulted in enzyme variants with two mutations on average. After cotransforming plasmids with the THP-specific biosensor and GfOMT1 into *E. coli* cells, the researchers then selected strains based on high fluorescence in the presence of the substrate NOR, indicating the production of THP. Strikingly, after only three rounds of directed evolution, variants could be identified that completely converted the precursor NOR to THP, thus demonstrating the effectiveness of the biosensor for metabolic engineering projects.

#### 3.5.5 Prerequisites for the successful directed evolution of aTFs

These examples highlight the significant progress that the combination of directed evolution and high-throughput fluorescence-based selection methods have created in the development of biosensors, but also show, as a recurring theme, the importance of reliable structural information about the aTF needed to accelerate the development process or even render the experimental effort feasible. This is especially true if an aTF needs to be engineered to recognize a non-cognate ligand for which there is no natural counterpart.

For the foreseeable future, experimental structure elucidation will progress at a much slower pace than the discovery of new sequence information, which emphasizes the need for structure-prediction methods that are reliable even in the absence of sufficient homology with solved structures.

Ideally, these efforts will lead to an automated protocol that hides the details of the modeling process from the synthetic biologist and automatically proposes mutagenesis libraries for a given objective.

## 4 Improving bioproduction using biosensors

aTF-based biosensors have a range of applications (e.g. diagnostics, environmental pollutant detection, biomaterials, health wearables…) (Moraskie et al., 2021). Arguably one of the most important applications is the use of biosensors in bioproduction. The world’s current production of chemicals is expected to double by 2030 (Nijman and Halpaap, 2019). Nevertheless, the production of materials, fuels, pharmaceuticals, fertilisers, foods, and other types of chemicals is still heavily reliant on traditional chemical synthesis based on unrenewable, polluting, fossil fuels (Naidu et al., 2021). Using microorganisms to substitute the chemical synthesis and move to greener, bio-based processes is a thriving field known as bioproduction (Zhang et al., 2017). The goal here is to improve the efficiency with which products are produced in comparison to traditional chemical approaches and to provide pathways for the production of novel compounds for which no synthetic routes exist. Bioproduction of chemicals and materials provides a renewable and economically viable alternative, easing the transition towards a circular “bioeconomy” (Cann, 2016). Next we detail two applications of biosensors that aim to improve bioproduction: screening and dynamic regulation.

### 4.1 aTF-based biosensors for screening

**FIGURE 4:**
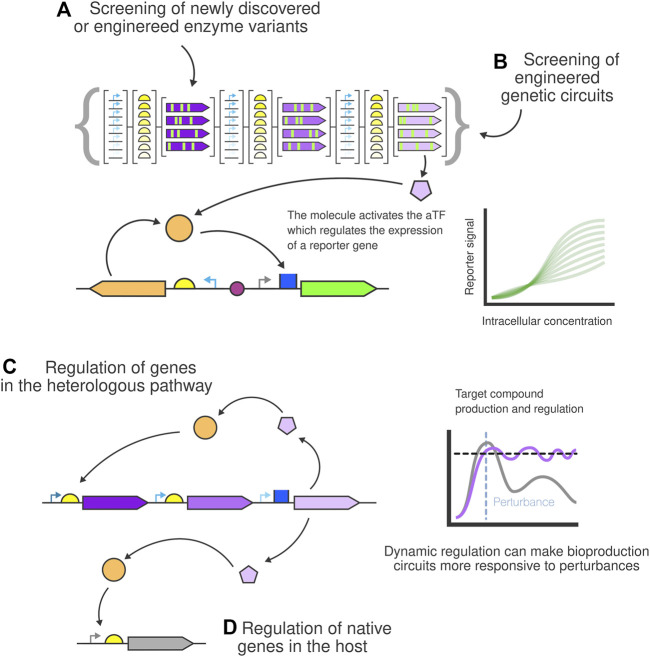
Bioproduction biosensor-based screening and dynamic regulation for bioproduction. New **(A)** enzyme variants and **(B)** genetic circuits can easily be screened for production by linking the production levels of the target metabolite to the output of the biosensor reporter. Dynamically regulated strains can be built using biosensors to control the production of the compound by regulating **(C)** the metabolic pathway and **(D)** native genes of the host.

One challenge preventing the broader adoption of current bioproduction strains is that they often perform poorly (low yield, slow production rate…) in industrial settings. Screening through hundreds of constructs and selecting the best performers is one of the most important bottlenecks. Thanks to advances in genetic engineering and automation, researchers can nowadays create hundreds of production strain variants in a short period of time. However, the next step involves screening those strains for production and yield. This can become a barrier since traditional methods like HPLC or LC-MS do not scale up easily. Biosensors can provide a simple, fast and affordable solution to the screening process of production strains by linking the biosensor output to the synthesis of the desired molecule (Kaczmarek and Prather, 2021). Biosensor-based screening can be used to screen for improved enzyme performance in a newly discovered or an engineered enzyme pool (Figure 4A) or for the screening of engineered genetic circuits of those enzymes and DNA parts (Figure 4B).

In order to identify biosensors with the appropriate sensitivity and dynamic range, several screening assays based on biosensors have been described. One of the simpler approaches involves the use of well plates and fluorescence detection to determine the production level of each variant (Yang et al., 2018; Zheng et al., 2018). Automation can increase the throughput of this assay by handling the library preparation, transformation and fluorescence measurement in each of the wells of the assay. Other approaches based on more advanced equipment [e.g. FACS (Liu et al., 2015), droplet-based screening (Siedler et al., 2017)] have also been tested. Tuning the dynamic range of biosensors *via* the modification of the promoter driving the expression of the reporter gene regulated by the aTF has also been proven possible (Chen et al., 2018). Biosensor mediated screening has also been described for large, multi-level CRISPRi experiments to fast-track genomic-level down-regulations that redirect the carbon flux towards the target metabolic route (Wang et al., 2023).

### 4.2 aTF-based biosensors for dynamic regulation

Engineered microorganisms often adapt poorly outside laboratory conditions, due to external factors originating from the conditions of production (e.g., large-scale bioreactors), such as pressure, acidity changes, accumulation of toxic metabolites, agitation, nutrient availability and heat transfer, among others (Wehrs et al., 2019). The strains are often unresponsive to external stimuli that may be present during industrial fermentation. This stress can cause undesired effects; for instance, the strain might stop producing the target compound by mutating or expelling the heterologous pathway (Wu et al., 2016). These adverse effects will lead to production processes that perform poorly when tested during the scale-up phase, thereby preventing the translation of many bioproduction projects into economically feasible industrial processes (Hartline et al., 2021).

Different approaches have been proposed to overcome those issues and increase the viability of large-scale cell-factory projects. Dynamic regulation is one of these strategies for controlling the production of key molecules, often found in nature and finely optimised through evolution. Similarly, biosensor-based dynamic regulation of microbial production pathways is a strategy that can be used to control genetic circuits based on a feedback loop that regulates the production of a target metabolite to keep its concentration at desired levels (Teng et al., 2022). The biosensor detects the presence of the metabolite and triggers the activation or inhibition of certain genes in the metabolic pathway (Figure 4C), making the system more responsive to possible detrimental conditions (Stevens and Carothers, 2015; Liu and Zhang, 2018; Hartline et al., 2021). To obtain the level of precise regulation required for some applications, simple single TF biosensors may not be enough. In this case, more complex circuits can be built, adding extra layers of complexity. For example, a plausible iteration would be to add another input molecule needed to trigger the desired reaction. This type of mechanism has been tested successfully with standard genetic regulator parts and inducers (Bordoy et al., 2019). Much more complex architectures are possible, mixing other common DNA parts (Nielsen et al., 2016). These approaches can be combined with native chromosomal gene regulation through direct control (Figure 4D) or by using CRISPRi (Huang et al., 2016; Wu et al., 2020) or antisense RNAs (Kim and Cha, 2003; Yang et al., 2018) that bind and repress native genetic pathways.

Growth-coupled production ties the target molecule (or one of its intermediate compounds) to the production/consumption of some essential cellular metabolite (Orsi et al., 2021). An example can be found in Wang et al. (2019) where the biosynthesis of pyruvate was limited to the heterologous pathway introduced into the cells. To accomplish this, the researchers deleted the native biosynthesis routes known to produce pyruvate. This essentially means that pyruvate will only be available as a byproduct of the heterologous pathway producing the target compound. In another example, Zhou and others (Zhou et al., 2021) built a 3-layer system that produces (2S)-naringenin, by regulating the essential compound malonyl-CoA. They used the TF FdeR, which is activated by the presence of (2S)-naringenin, and PadR TF, which is activated by *p*-coumaric acid, as feedback regulators of the (2S)-naringenin pathway. Initial low concentrations of (2S)-naringenin allow malonyl-CoA to be used in fatty acid (FA) biosynthesis pathways, favouring cell growth. Higher concentrations of naringenin, represses these FA synthesis routes, slowing cell growth and increasing the availability of malonyl-CoA for the production of more (2S)-naringenin. Through several rounds of optimization, including directed evolution of biosensors and optimization of fermentation conditions, titers above 500 mg/L from glucose were obtained in 5-L bioreactors using an *E. coli* chassis. Other strategies decouple the production phase from the growth phase of the microbial culture. For example, to improve the production of glucaric acid (GA), the glycolysis pathway can be repressed using the accumulation of *N*-Acyl homoserine lactone (AHL) in *E. coli* (Doong et al., 2018). Pyruvate-responsive circuits were also built in *Bacillus subtilis* for the regulation of glucaric acid production (Xu et al., 2020). Pyruvate induces the expression of the GA pathway and suppresses glycolysis. Finally, malonyl-CoA, another essential cellular metabolite, can be used as the key regulator. In (Xu et al., 2014), Xu et al. built a regulation circuit that activates the synthesis of malonyl-CoA and represses the fatty acid synthesis pathway that consumes it when the compound is in low concentrations and *vice versa*.

Even though the aforementioned examples show that successful dynamic regulation is possible, true industrial utilisation of dynamic regulation circuits and scale-up is still rare in bioproduction projects. Tested under homogeneous laboratory conditions, dynamic regulation bioproduction circuits are not normally designed to react to environmental changes and, therefore, the resulting strains adapt poorly to the different conditions during the scale-up process (Neubauer and Junne, 2010). Among other reasons we can include the lack of functional and well-characterised biosensor circuits against a wider range of target molecules and the inherent difficulty of dynamically controlling complex cellular, enzymatic, genetic and molecular networks which can show unwanted crosstalk between different components. In order to address these shortcomings, a different type of regulation relies on automatically reacting to these conditions and controlling the production accordingly (Boada et al., 2020).

## 5 Conclusions and future perspectives

In this work we reviewed recent advances in the prediction, design and validation of biosensor circuits focused on aTFs and whole-cell implementations. This type of biosensor can be used to detect a wide range of molecules (e.g., ions, sugars, drugs, hormones…) for different uses, including environmental, medical and industrial applications. We showcased the role of biosensors in the development of new-generation bioproduction devices, driving forward important steps for bioproduction advancement such as screening and dynamic regulation of producer strains. aTF biosensors, however, suffer from certain shortcomings that will need addressing in future research endeavours. To that end, we detail some of the possible routes towards better biosensors.

As described in this article, several methodologies can be used to reveal as-yet-undiscovered aTFs that may behave closely to what a biosensor designer might expect. However, associated tools and databases only offer fragmented information and it is up to the designer to gather and trust the results from different sources. Therefore, a single computational tool that takes this burden from the researcher and provides confidence metrics is needed. Even though directed mutagenesis has shown great potential to improve the performance of a given aTF, it is still not possible to accurately predict the impact of a mutagenesis experiment on the biosensor characteristics (dynamic range, operational range…). Structural approaches are probably the key to this issue and the recent development of structural prediction and docking programs may soon provide researchers with this type of tool (Mullard, 2021). Until recently, structure-based design was simply impossible in the absence of any relevant homology. With the advent of AI-based approaches to protein-structure prediction [e.g., AlphaFold (Jumper et al., 2021)]—The success stories of which suggest that *in silico* models at almost atomic resolution might be within reach—It might be possible to take the computational design of bespoke binding sites from merely instructing mutagenesis experiments on an, at best, semi-quantitative basis, to predictive reliability.

As shown for several applications in this review, biosensor circuits can be used to screen for the best performers in a bioproduction experiment by linking the reporter expression to the production level. High-throughput FACS or the less-scalable but more affordable plate-reader-based approach can be used to select the cells with higher reporter expression levels. However, this can become a problem if so-called cheaters come into play (Trivedi et al., 2022), giving high reporter signals without showing high production rates of the target compound. More methods that address this issue are needed. Finally, the most important future milestone for the field would be to develop a toolbox that can predict and model—Within the accuracy levels required for the application—The behaviour of a biosensor for a given design and target molecule, while taking process conditions and possible environmental perturbations into account that may appear during the experiment.
